# Styrone

**Published:** 1891-06-27

**Authors:** 


### THERAPEUTICS.

### STYRONE.

Two forms of styrone are met with: the one consists of white needle-like crystals, which are readily soluble in alcohol or ether; the other and cheaper form being an oily yellowish fluid, with a somewhat bitter taste. The latter is slightly soluble in water, but is, like the other form, readily dissolved by ether or alcohol, imparting to the solution a very pleasant aromatic odour. This latter form has been recommended by Dr. Cheltsoff in chronic otitis media, and our own experience of this remedy bears out the good opinion he has formed of its efficacy. Dr. Cheltsoff uses a solution of styrone in alcohol diluted with water to the strength of 1 in 500, with which the meatus should be syringed carefully two or three times a day. We have never found any disagreeable effects from a 1 per cent. solution, although the weaker seems equally efficacious. It is strongly disinfectant and deodorant, and we have found great improvement in several cases of ozoena in which it has been used. It is much less irritating than the majority of disinfectant fluids are to the nasal mucous membrane; it appears to be anæsthetic to a slight extent, a fact of considerable importance for rhinological purposes. In one very troublesome and long-standing case of otitis media, we found great improvement from the use of the solution.

